# Housekeeping genes essential for pantothenate biosynthesis are plasmid-encoded in *Rhizobium etli *and *Rhizobium leguminosarum*

**DOI:** 10.1186/1471-2180-11-66

**Published:** 2011-04-05

**Authors:** Tomás Villaseñor, Susana Brom, Araceli Dávalos, Luis Lozano, David Romero, Alejandro García-de los Santos

**Affiliations:** 1 Programa de Ingeniería Genómica, Centro de Ciencias Genómicas, Universidad Nacional Autónoma de México, Apdo. Postal 565-A. Cuernavaca, Morelos, México; 2 Programa de Genómica Evolutiva, Centro de Ciencias Genómicas, Universidad Nacional Autónoma de México, Apdo. Postal 565-A. Cuernavaca, Morelos, México

## Abstract

**Background:**

A traditional concept in bacterial genetics states that housekeeping genes, those involved in basic metabolic functions needed for maintenance of the cell, are encoded in the chromosome, whereas genes required for dealing with challenging environmental conditions are located in plasmids. Exceptions to this rule have emerged from genomic sequence data of bacteria with multipartite genomes. The genome sequence of *R. etli *CFN42 predicts the presence of *panC *and *panB *genes clustered together on the 642 kb plasmid p42f and a second copy of *panB *on plasmid p42e. They encode putative pantothenate biosynthesis enzymes (pantoate-β-alanine ligase and 3-methyl-2-oxobutanoate hydroxymethyltransferase, respectively). Due to their ubiquitous distribution and relevance in the central metabolism of the cell, these genes are considered part of the core genome; thus, their occurrence in a plasmid is noteworthy. In this study we investigate the contribution of these genes to pantothenate biosynthesis, examine whether their presence in plasmids is a prevalent characteristic of the *Rhizobiales *with multipartite genomes, and assess the possibility that the *panCB *genes may have reached plasmids by horizontal gene transfer.

**Results:**

Analysis of mutants confirmed that the *panC *and *panB *genes located on plasmid p42f are indispensable for the synthesis of pantothenate. A screening of the location of *panCB *genes among members of the *Rhizobiales *showed that only *R. etli *and *R. leguminosarum *strains carry *panCB *genes in plasmids. The *panCB *phylogeny attested a common origin for chromosomal and plasmid-borne *panCB *sequences, suggesting that the *R. etli *and *R. leguminosarum panCB *genes are orthologs rather than xenologs. The *panCB *genes could not totally restore the ability of a strain cured of plasmid p42f to grow in minimal medium.

**Conclusions:**

This study shows experimental evidence that core *panCB *genes located in plasmids of *R. etli *and *R. leguminosarum *are indispensable for the synthesis of pantothenate. The unusual presence of *panCB *genes in plasmids of *Rhizobiales *may be due to an intragenomic transfer from chromosome to plasmid. Plasmid p42f encodes other functions required for growth in minimal medium. Our results support the hypothesis of cooperation among different replicons for basic cellular functions in multipartite rhizobia genomes.

## Background

Multipartite genomes are common among members of the α-proteobacteria [1]. Most symbiotic nitrogen-fixing bacteria belonging to the genera *Rhizobium*, *Sinorhizobium, Mesorhizobium *and *Bradyrhizobium *possess multipartite genomes organized as a single circular chromosome and a variable number of large plasmids [2]. In some species plasmids can represent, in terms of size, up to 40% of the total genome. In *Rhizobium *and *Sinorhizobium *species one plasmid (pSym) concentrates most of the genes required for nodulation and nitrogen fixation [3]. The complete genome sequences of different rhizobia have revealed that plasmids harbor mainly accessory genes and that most encode predicted transport systems and a variety of catabolic pathways that may contribute to the adaptation of rhizobia to the heterogeneous soil and nodule environments [2,4]. These genes are absent from closely related genomes, lack synteny and their G+C composition differs from that of the core genes. The core genes are mainly located on chromosomes, have essential functions in cell maintenance and have orthologs in related species [5,6]. In spite of this evidently biased distribution of core genes in the chromosome and accessory genes in plasmids, it is important to highlight the fact that there are interesting exceptions to this genomic rule: several typical core genes have been found encoded on rhizobia plasmids. Some are copies of genes located on chromosomes, with redundant functions that are totally dispensable for normal growth. Examples of these genes are the multiple copies of chaperonin-encoding genes *groEL/groES *[7,8], two *tpiA *genes encoding putative triose phosphate isomerase, a key enzyme of central carbon metabolism [4,6,9], and two putative *S. meliloti *asparagine synthetases (*asnB *and *asnO*), which may have a role in asparagine synthesis from aspartate by ATP-dependent amidation [10]. In contrast to these reiterated genes, a few single copy core genes have also been localized in plasmids. The *tRNA *specific for the second most frequently used arginine codon, CCG, is located on pSymB in *S. melioti *[10]. Since this gene lies within a region of pSymB that could not be deleted [11], it is assumed to be essential for cell viability. The single copy of the *minCDE *genes, conceivably involved in proper cell division, have also been found in plasmids of *S. meliloti, R. leguminosarum *and *R. etli *[4,6,10]. Studies in *S. meliloti *have demonstrated that even though these genes are expressed in free-living cells and within nodules they are nonessential for cell division, since their deletion did not produce the small chromosomeless minicells observed in *E. coli *and *Bacillu subtilis *[12].

A recent bioinformatic study revealed that approximately ten percent of the 897 complete bacterial genomes available in 2009 carry some core genes on extrachromosomal replicons [13]. However, very few of these genes have been functionally characterized and so their real contribution to bacterial metabolism is still an open question.

The complete genome sequence of *R. etli *CFN42 predicts that two putative "housekeeping" genes, *panC *and *panB*, which may be involved in pantothenate biosynthesis, are clustered together on plasmid p42f. Pantothenate is an essential precursor of coenzyme A (CoA), a key molecule in many metabolic reactions including the synthesis of phospholipids, synthesis and degradation of fatty acids, and the operation of the tricarboxylic acid cycle [14]. The *R. etli panC *gene is predicted to encode the sole pantoate-β-alanine ligase (PBAL), also known as pantothenate synthetase (PS) (EC 6.3.2.1), present in the *R. etli *genome. The function of this enzyme is the ATP-dependent condensation of D-pantoate with β-alanine to form pantothenate, the last step of the pantothenate biosynthesis pathway. The *panB *gene encodes the putative 3-methyl-2-oxobutanoate hydroxymethyltransferase (MOHMT) (EC 2.1.2.11), also known as ketopantoate hydroxymethyltransferase (KPHMT), the first enzyme of the pathway, responsible for the formation of α-ketopantoate by the transfer of a methyl group from 5,10-methylentetrahydrofolate to alpha-ketoisovalerate. The complete genome sequence of *R. etli *CFN42 predicts that a second putative MOHMT enzyme (RHE_PE00443), similar to the product of *panB*, is encoded on plasmid p42e.

In this work we describe the isolation and use of *panC *and *panB *mutants to analyze the involvement of these plasmid-encoded genes in pantothenate biosynthesis. A survey of the localization of *panCB *genes among members of the *Rhizobiales *with multipartite genomes allowed us to infer a *panCB *phylogeny and to establish the probable chromosomal origin of these plasmid-borne genes. We also report that the *panCB *genes could not totally restore the growth in minimal medium (MM) of a strain cured of plasmid p42f, suggesting that other functions essential for growth in MM are encoded in this plasmid.

## Results

### Functional characterization of plasmid p42f encoded *panCB *genes

**Figure 1:**
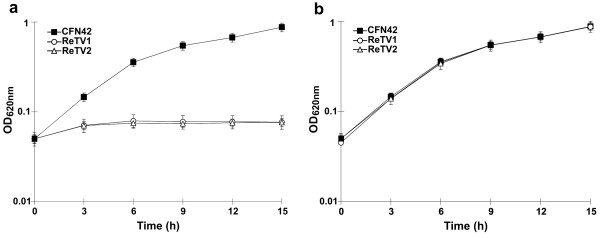
**Pantothenate auxotrophy of *R. etli *CFN42 *panC *and *panB *mutants**. Growth of the *R. etli *CFN42 wild-type strain and its derivative *panC *(ReTV1) and *panB *(ReTV2) mutants in: (a) minimal medium, (b) minimal medium supplemented with 1 μM calcium pantothenate. Values represent the means of three independent experiments; error bars show standard deviations.

The predicted function of the product of *panC *(RHE_PF00001) annotated as PBAL, is the catalysis of the last step of pantothenate synthesis. This PBAL (298 amino acids) showed 43% identity and 62% similarity over 279 amino acids with the functionally characterized PBAL of *E. coli *K12 (284 amino acids). A search for conserved domains (CD-search) at NCBI-CDD revealed the presence of a typical pantoate-binding site. The *panB *gene (RHE_PF00002) is located immediately downstream of *panC*. The four nucleotide overlap between the *panC *TGA codon and *panB *ATG codon suggest that these genes might be transcribed as an operon. The *panB *gene encodes a putative MOHMT, the first enzyme of the pantothenate pathway. A BlastP comparison between the functionally characterized MOHMT of *E. coli *K12 (264 amino acids) and the putative MOHMT encoded on plasmid p42f of *R. etli *CFN42 (273 amino acids) showed 37% identity and 56% similarity over a length of 240 amino acids. A CD-search indicated that in the putative MOHMT of *R. etli *CFN42 the magnesium binding and active site domains are conserved. Additionally, Paralog Search (KEGG SSDB) and pathway tools predicted a second probable MOHMT, encoded on plasmid p42e (locus tag RHE_PE00443). Both proteins are similar in length (273 and 270 aa for the products encoded by *panB *and RHE_PE00443, respectively). However, a BlastP comparison of these sequences showed only 36% identity and 56% similarity over a tract of 140 amino acids. A CD-search revealed that only 5 of 12 of the invariable residues present in the active site domain are conserved in RHE_PE00443. The metal binding domain could not be detected by the CD-search. To determine whether the *panC *and *panB *genes located on plasmid p42f are required for pantothenate synthesis, mutations in these genes were generated by site-directed vector integration mutagenesis via a single cross-over recombination (see details in Material and Methods and Table 1). Mutants ReTV1 (*panC *^-^) and ReTV2 (*panB^-^*) were unable to grow in minimal medium (MM) lacking calcium pantothenate (Figure 1a). Supplementation of MM with 1 μM calcium pantothenate allowed the *panC *and *panB *mutants to recover their wild-type growth rate (Figure 1b). The pantothenate auxotrophy displayed by the *panB *mutant ReTV2 allowed us to discard a functional role of the putative MOHMT encoded by RHE_PE00443 in pantothenate biosynthesis. Moreover, a pBBRMCS3 clone constitutively expressing RHE_PE00443 (pTV7) was unable to complement the pantothenate auxotrophy of the *panB *mutant (data not shown).

**Table 1:** Bacterial strains and plasmid.

Strain or plasmid	Relevant genotype	Reference or source
***Rhizobium etli***		
CFN42	Wild type; Nal^r^	[6]
ReTV1	CFN42 *panC*::pTV1; Km^r^	This study
ReTV1-4	CFN42 *panC*::pTV1 complemented with pTV4; Tcr Km^r ^	This study
ReTV1-5	CFN42 *panC*::pTV1 complemented with pTV5; Tcr Km^r^	This study
ReTV2	CFN42 *panB*::pTV2; Km^r^	This study
ReTV2 -4	CFN42 *panB*::pTV2 complemented with pTV4; Tc^r ^Km^r^	This study
ReTV2 -6	CFN42 *panB*::pTV2 complemented with pTV6; Tc^r ^Km^r^	This study
ReTV2 -7	CFN42 *panB*::pTV2 complemented with PTV7; Tc^r ^Km^r^	This study
ReTV3	CFN42 *argE*::pTV3; Km^r^	This study
CFNX186	CFN42 cured of plasmid p42f; Nal^r^	[18]
CFNX186-4	CFNX186 complemented with pTV4; Tc^r^	This study
CFNX186-24	CFNX186 complemented with pCos24; Tc^r^	[30]
CIAT 652	Wild type; Nal^r^	[38]
CIAT 894	Wild type; Nal^r^	[38]
Kim5	Wild type; Nal^r^	J. Handelsman, University of Wisconsin, MD
IE4771	Wild type; Nal^r^	[15]
***Escherichia coli***		
DH5α	Host for recombinant plasmids; Nal^r^	Stratagene
S17-1	C600::RP4-2(Tc::Mu) (Km::Tn7) Donor for conjugation	[39]
**Plasmids**		
pBC	pBluescript II SK(+) phagemid vector; Cm^r^	Stratagene.
pK18mob	pK18, derivative mob; Km^r^	[29]
pRK7813	Broad-host-range cosmid vector; Mob, IncP, Tc^r^	[40]
pBBRMCS3	Broad-host-range cloning vector; Mob; Tc^r^	[41]
pBC1	pBC harboring a 400-bp *BamH*I-*Xba*I PCR fragment of *panC*; Cm^r ^	This study
pBC2	pBC harboring a 400-bp *BamH*I-*Xba*I PCR fragment of *panB*; Cm^r ^	This study
pTV1	pK18mob harboring a 400-bp *KpnI*-*Xba*I PCR fragment of *panC*; Km^r ^	This study
pTV2	pK18mob harboring a 400-bp *KpnI*-*Xba*I PCR fragment of *panB*; Km^r ^	This study
pTV3	pK18mob harboring a 400-bp *KpnI*-*Xba*I PCR fragment of *argE*; Km^r^	This study
pTV4	pRK7813 harboring a 3.1 kb *EcoRI *fragment of pCos24 containing *panC *and *panB*; Tc^r^	This study
pTV5	pBBRMCS3 harboring a 1.2 kb *KpnI*-*Xba*I PCR fragment containing *panC*; Tc^r ^	This study
pTV6	pBBBRMCS3 harboring a 1 kb *KpnI*-*Xba*I PCR fragment containing *panB*; Tc^r ^	This study
pTV7	pBBRMCS53 harboring a 1 kb *KpnI*-*Xba*I PCR fragment containing RHE_PE00443; Tc^r^	This study
pcos24	20 Kb EcoRI fragment of plasmid p42f cloned in pLAFR1 containing *panC*, *panB*, *oxyR *and *katG*; Tc^r^	[30]

Plasmid pTV4, harboring the *panC *and *panB *genes, as well as plasmids pTV5 and pTV6, carrying only *panC *or *panB *respectively, were introduced into mutant strains ReTV1 and ReTV2 and the growth phenotype of each construction was evaluated in MM. The *panC *mutant ReTV1 complemented with the *panCB *genes (ReTV1-4) recovered wild type growth in MM. In contrast, when complemented only with *panC *(strain ReTV1-5) no growth occurred in the absence of pantothenate. These results strongly suggest that the *panCB *genes form a single transcriptional unit. As expected, wild type growth of *panB *mutant ReTV2 was recovered by complementation with the *panCB *genes or with the *panB *gene (strains ReTV2-4 and ReTV2-6 respectively).

### The occurrence of *panCB *genes in plasmids is highly conserved among *R. etli *and *R. leguminosarum *strains but not in other members of the *Rhizobiales *with multipartite genomes

To investigate whether the presence of the *panCB *genes in plasmids is a common characteristic of the *Rhizobiales*, we examined the location of *panCB *genes in 22 members of the *Rhizobiales *having fully sequenced multipartite genomes (Table 2). To date, the genomes of seven *R. etli *strains, in addition to CFN42, have been totally sequenced [15]. However, with the exception of strain CIAT 652, the genomes were released as draft assemblies, precluding *panCB *localization. We experimentally determined the localization of *panCB *genes in the genome of four of these *R. etli *strains (CIAT 894, Kim5, 8C-3, and IE4771) by hybridization of their plasmid profiles with [^32^P]dCTP-labelled *panC *and *panB *genes from CFN42 under high stringency conditions. Both probes produced intense hybridization signals on the same plasmid of each strain, indicating that the *panCB *genes are also plasmid-borne in these *R. etli *strains (Table 2). Coincidentally, in the three *R. leguminosarum *strains with fully sequenced genomes reported in the NCBI database, the *panCB *genes are assigned to plasmids. In contrast, in other species of *Rhizobiales *with multipartite genomes, the *panCB *genes are always confined to the chromosome, or to chromosome I in those species harboring two chromosomes, with exception of *Agrobacterium tumefaciens *C58 which carries *panCB *on the linear chromosome II and *Methylobacterium nodulans *ORS2060 that carries *panC *on their single chromosome and *panB *on plasmid pMNOD02 (Table 2).

**Table 2:** Localization of the *panCB *genes in representative members of the *Rhizobiales *with multipartite genomes.

Strain			Localization of	
	Genome number Chr	Structure of Plasmids	*panC*	*panB*
*Brucella abortus *bv. 1 str. 9-941	2	0	ChrI	ChrI
*B. melitensis *16M	2	0	ChrI	ChrI
*B. ovis *ATCC 25840	2	0	ChrI	ChrI
*Sinorhizobium meliloti *1021	1	2	Chr	Chr
*S. medicae *WSM419	1	3	Chr	Chr
*Ochrobactrum anthropi *ATCC 49188	2	4	ChrI	ChrI
*Agrobacterium radiobacter *K84	2	3	ChrI	ChrI
*A. vitis *S4	2	5	ChrI	ChrI
*A. tumefaciens *C58	2	2	ChrII	ChrII
*Rhizobium etli *CFN42	1	6	p42f	p42f
*R. etli *CIAT 652	1	3	pc	pc
*R. etli *CIAT 894*	1	4	pd	pd
*R. etli *Kim5*	1	4	pc^†^/pd^† ^	pc^†^/pd^† ^
*R. etli *IE4771*	1	4	pd	pd
*R. etli *8C-3*	1	3	pc	pc
*R. leguminosarum *bv. viciae 3841	1	6	pRL12	pRL12
*R. leguminosarum *WSM1325	1	5	pR132501	pR132501
*R. leguminosarum *WSM2304	1	4	pRLG201	pRLG201
*Rhizobium *sp. NGR234	1	2	Chr	Chr
*Mesorhizobium loti *MAFF303099	1	2	Chr	Chr
*M. sp*. BNC1	1	3	Chr	Chr
*Methylobacterium extorquens *AM1	1	4	Chr	Chr
*M. radiotolerans *JCM2831	1	8	Chr	Chr
*M. nodulans *ORS2060	1	7	Chr	pMNOD2
*Bradyrhizobium sp*. BTAi1	1	1	Chr	Chr
*Nitrobacter hamburgensis *X14	1	3	Chr	Chr
*Xantobacter autotrophicus *Ry2	1	1	Chr	Chr

Abbreviations are as follows: Chr, chromosome of those *Rhizobiales *with one chromosome; Chr I and Chr II, chromosome I and chromosome II respectively in those *Rhizobiales *harboring two chromosomes; p, plasmid. *Rhizobium species in which localization of *panCB *genes was done by Southern blot hybridization of plasmid profiles. ^†^Plasmids with very similar electrophoretic mobility gave as result ambiguous plasmid localization of *panC *and *panB *homologous sequences.

### Phylogenetic analysis of rhizobial *panCB *genes indicates a common origin of chromosomal and plasmid-borne sequences

**Figure 2:**
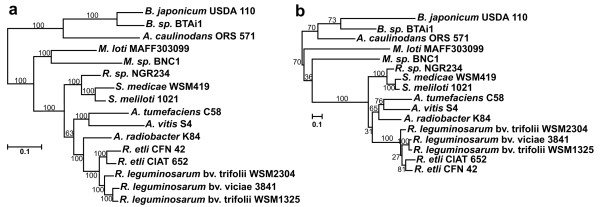
**Comparison of phylogenetic trees constructed from core and *panCB *genes**. Maximum-likelihood phylogenetic trees of 16 *Rhizobiales *constructed using the concatenated nucleic acid sequences of 10 housekeeping genes (a) or *panC *and *panB *concatenated genes (b). Bootstrap values are shown over each branch (based on 100 pseudo-replicates).

Two possible hypotheses were considered to explain the presence of *panCB *genes in plasmids of *R. etli *and *R. leguminosarum *strains: (1) an intragenomic rearrangement of *panCB *genes from chromosome to plasmid, which must have occurred in the last common ancestor of both species; (2) by xenologous gene displacement, that is, a horizontal transfer event in which a gene is displaced by a horizontally transferred ortholog acquired from another lineage [16]. In the latter hypothesis we assume that the presence of these xenolog genes in plasmids conferred a selective advantage that may have eventually led to the loss of the chromosome-located *panCB *genes. To test these hypotheses the phylogeny of 16 rhizobial species inferred from ten orthologous single copy housekeeping genes (*fusA, guaA, ileS, infB, recA, rplB, rpoB, rpoC, secY and valS*) located on primary chromosomes, was compared with the phylogeny of the same rhizobial species inferred from the *panCB *genes located on plasmids and chromosomes. The rationale for this comparison was that if the plasmid-borne *panCB *phylogeny agrees with the current phylogeny of the *Rhizobiales*, inferred from the housekeeping genes, it would support the hypothesis of intragenomic transfer of the *panCB *genes. On the other hand, if both phylogenies are incongruent, it would favor the hypothesis of horizontal transfer of the *panCB *genes. Concatenated nucleic acids multiple alignments were used to infer both phylogenies with the maximum likelihood method described in materials and methods. The resulting phylogenetic trees are shown in Figure 2. The housekeeping genes inferred tree (Figure 2a) was consistent with the recently reported phylogeny of 19 *Rhizobiales *performed on a data set of 507 homologous proteins from the primary chromosome [17]. Both trees are in close agreement with the phylogeny inferred from the *panCB *genes (Figure 2b). Thus the phylogeny of *R. etli *and *R. leguminosarum *inferred from plasmid-encoded *panCB *genes is consistent with the phylogeny deduced from their housekeeping genes supporting the hypothesis of a chromosomal origin for the plasmid-encoded *panCB *genes.

### The *panCB *genes do not fully complement the growth deficiency of a *R. etli *CFN42 p42f cured derivative in MM

**Figure 3:**
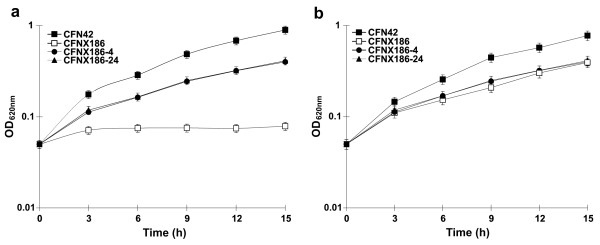
***panCB *genes do not fully restore the growth deficiency of CFNX186**. Growth of *R. etli *CFN42 wild-type strain, its p42f-cured derivative CFNX186, CFNX186 complemented with the *panCB *genes (CFNX186-4) and CFNX186 complemented with a 20 kb *EcoR*I fragment of plasmid p42f containing the *panC*, *panB*, *oxyR *and *katG *genes (CFNX186-24) in: (a) minimal medium, (b) minimal medium supplemented with 1 μM pantothenate. Growth curves are the mean of at least three independent experiments; error bars represent standard deviations.

It was reported previously that *R. etli *CFNX186, a p42f-cured derivative of *R. etli *CFN42, is unable to grow in MM [18]. To assess if the growth deficiency of strain CFNX186 in MM was due to the absence of the *panC *and *panB *genes, plasmid pTV4 (*panCB*) was introduced into strain CFNX186. The growth of the transconjugant (CFNX186-4) after 15 hours of culture in MM was only 50% that of the WT strain grown under the same conditions (Figure 3a). The growth of CFNX186-4 did not improve even after 72 h in culture (data not shown). Interestingly, strain CFNX186-4 had the same growth rate as strain CFNX186 cultured in MM supplemented with 1 μM calcium pantothenate (Figure 3b). This shows that the growth deficiency of CFNX186 is only partly due to the absence of the *panCB *genes and indicates that other functions encoded in plasmid p42f are required for growth in MM.

Previous studies have demonstrated that the *katG *gene, which encodes the sole catalase-peroxidase expressed in free-living growth conditions, is located on plasmid p42f of *R. etli *CFN42. These studies also revealed that the growth rate of a *katG *mutant in MM was significantly reduced in comparison with that of the wild-type parental strain [19]. On plasmid p42f *katG*, as well as its putative transcriptional regulator protein encoded by *oxyR*, are located 80 bp downstream of the *panCB *genes. We speculated that introduction of the *panCB *genes together with the *katG *and *oxyR *genes might improve the growth of CFNX186 in MM. To test this hypothesis, we used pCos24, which contains a 20 kb fragment of p42f carrying *panCB*, *katG *and *oxyR *(see Material and Methods). pCos24 was introduced into CFNX186 and the resulting transconjugant (CFNX186-24) grown in MM. Figure 3 shows that after 15 hours of culture there was no significant difference between the growth rate of CFNX186 complemented only with *panCB *(CFNX183-4), and CFNX186 complemented with cosmid pCos24 (CFNX186-24). Furthermore, the growth of CFNX186-24 did not increase even after 72 h of culture (data not shown) indicating that *katG *and *oxyR *did not improve the growth rate of *panCB *complemented CFNX186 in MM. We also tested the possibility that arginine might improve the growth of strain CFNX186-24 due to the presence of a putative *N*-acetylornithinase (EC 3.5.1.16) encoded in the plasmid p42f. In the *Enterobactericeae *this enzyme catalyzes the conversion of *N*-acetylornithine to ornithine, a key step in the arginine biosynthesis pathway [20]. However, the growth deficiency of strain CFN186-24 in MM was not corrected by the addition of 1, 5, 10 or 15 mM arginine (data not shown). Furthemore, we constructed an *argE *mutant strain (ReTV3, Table 1) that was able to grow in MM without exogenous arginine at the same rate as parental strain CFN42 (data not shown), confirming that this gene is not essential for arginine synthesis.

## Discussion

Seminal studies on the phenotypic characterisation of plasmid-cured strains of *R. leguminosarum *and *R. etli *revealed that the absence of several plasmids cause a growth deficiency in rich and minimal medium [18,21]. These findings suggested that undefined metabolic traits are present on rhizobial plasmids. The bioinformatic analysis of 897 bacterial genomes performed by Harrison *et al *[13] revealed the presence of extrachromosomal core genes in 82 genomes mainly belonging to the Proteobacteria. In contrast with these *in silico *data, there is little experimental information on the contribution of these core genes to bacterial metabolism or cellular process. The few genes that have been functionally characterized encode redundant functions and are totally dispensable for the cell [7-9,12]. Our study provides experimental evidence that the enzymes MOHMT (EC 2.1.2.11) and PBAL (EC 6.3.2.1) encoded on plasmid p42f are indispensable for the synthesis of pantothenate. Moreover, our results showed that the cluster of *panCB, katG *and *oxyR *genes was insufficient to restore full growth capacity to the p42f cured derivative CFNX186, implying that in addition to pantothenate synthesis, there are more functions encoded on plasmid p42f required for growth in MM. Obvious candidates for these functions could not be identified *a priori *among the 567 proteins encoded in p42f even though their predicted functions were recently updated with KAAS (KEGG Automatic Annotation Server and Pathway Reconstruction Server). We discarded arginine limitation as the cause for the growth deficiency of strain CFNX186-24. The arginine prototrophy displayed by a mutation in the p42f encoded *argE *suggests that in *R. etli *the conversion of *N*-acetylornithine to ornithine is catalyzed by the chromosome-encoded ArgJ, an ornithine acetyltransferase (OATase, EC 2.3.1.35), which transfers the acetyl group of *N*-acetylornithine to glutamate to produce ornithine and *N*-acetylglutamate. Functional OATases have been found in the majority of bacteria [20].

Also, we have demonstrated that plasmid-localization of *panCB *in *R. etli *CFN42 is not unique to this strain. A screening of the location of *panCB *genes among members of the *Rhizobiales*, showed that the occurrence of these genes in plasmids is a highly conserved trait among *R. etli *and *R. leguminosarum *strains. Furthermore, the synteny of the *panCB*, *oxyR*, *katG *genes in *R. etli *CFN42 is conserved in *R. etli *CIAT652 and in *R. leguminosarum *strains 3841, WSM1325 and WSM2304. In contrast, genomes of *Rhizobium sp., Sinorhizobium, Bradyrhizobium *and *Mesorhizobium *species carried chromosomal *panCB *genes. Only in *A. tumefaciens *C58 the *panCB *genes are localized in the linear chromosome, whereas in all other *Rhizobiales *harboring secondary chromosomes the *panCB *genes were located in chromosome I. A bioinformatic analysis with MicrobesOnline operon predictions [22] indicates that *panCB *genes are organized as possible operons in most of the *Rhizobiales *examined in this work: all these predicted operons conserve the four nucleotide overlap between the *panC *TGA codon and the *panB *ATG codon observed in *R. etli *CFN42 (data not shown). In the genomes of *Bradyrhizobium sp*. BTAi1, *Nitrobacter hamburgensis *X14, *Methylobacterium extorquens *AM1, *Methylobacterium radiotolerans *JCM2831 and *Xantobacter autotrophicus *Ry2, *panC *and *panB *are encoded in separate chromosomal loci, whereas in *Methylobacterium nodulans *ORS2060 *panC *is located in the chromosome and *panB *in plasmid pMNOD02.

The *Rhizobiales *phylogeny inferred from concatenated *panC *and *panB *genes was consistent with the phylogeny deduced from 10 concatenated housekeeping genes. The low bootstrap values obtained for some nodes of the *panCB *phylogeny might be due to the small number of informative characters in the alignments of only two genes (1 977 nucleotides). This is consistent with previous reports that state that trees from longer alignments obtained by the concatenation of genes encoding multiple-protein families have higher bootstrap support than trees inferred from genes encoding single proteins [23]. The phylogenetic relationships among *Rhizobium *species carrying *panCB *genes in plasmids with their closest relatives, *Agrobacterium *and *Sinorhizobium *species, harboring *panCB *genes in the chromosome was also observed in neighbor-joining trees inferred from single *panC *and *panB *genes (data not shown). These data agree with the hypothesis that plasmid-encoded *panCB *genes are orthologs of the *panCB *genes located in chromosome. From these results, we propose that the presence of the *panCB *genes in plasmids in *R. etli *and *R. leguminosarum *species may be due to an intragenomic transfer event from chromosome to plasmid. The mechanism leading to the transfer of core genes from chromosome to plasmids could involve cointegration and excision events between the replicons, similar to rearrangements that have been visualized in *S. meliloti *[24]. The translocation of genes from chromosome to plasmids may be part of the complex evolution of multipartite genomes. A study based on the analysis of clusters of syntenic genes shared among plasmids and secondary chromosomes of bacteria with multipartite genomes suggested that secondary chromosomes may have originated from an ancestral plasmid to which genes had been transferred from a primary chromosome [17].

Our pioneering work on plasmid-encoded functions in *R. etli *CFN42 established that a functional relationship among different replicons is required for symbiotic and free-living functions [18,25]. More recently, a functional connectivity among most of the proteins encoded in the replicons of *R. etli *CFN42 was predicted *in silico *[6]. Our results demonstrated that the putative MOHMT encoded by RHE_PE00443 is not functional under the conditions studied and provides evidence of functional cooperation between p42f and chromosomally encoded proteins for pantothenate biosynthesis.

## Conclusions

Our study shows that the presence of the core *panCB *genes in a plasmid is a characteristic conserved in *R. etli *and *R. leguminosarum *strains but not in other *Rhizobiales*. The phylogenetic approach used in this study suggests that the unusual presence of *panCB *in plasmids may be due to an intragenomic transfer event from chromosome to plasmid rather than a xenologous gene displacement. Using *R. etli *CFN42 as a model, we showed that the plasmid-encoded core *panCB *genes were indispensable for the synthesis of pantothenate. The *panCB *genes could not totally restore growth of a strain cured of plasmid p42f in minimal medium, suggesting that other functions essential for growth in this medium are encoded in this plasmid. Our results support the hypothesis of functional cooperation among different replicons for basic cellular functions in multipartite rhizobial genomes.

## Methods

### Bacterial strains, media and growth conditions

The bacterial strains and plasmids used are listed in Table 1. *Rhizobium *strains were grown at 30°C in three different media: a) PY rich medium [26], b) Minimal medium (MM) [27] and c) Minimal medium plus 1 μM calcium pantothenate (MMP). MM was prepared as follows: a solution containing 10 mM succinate as carbon source, 10 mM NH_4_Cl as nitrogen source, 1.26 mM K_2_HPO_4_, 0.83 mM MgSO_4_, was adjusted to pH 6.8 and sterilized. After sterilization the following components were added to the final concentration indicated: 0.0184 mM FeCl_3 _6H_2_O (filter sterilized), 1.49 mM CaCl_2 _2H_2_O (autoclaved separately), 10 μg ml^-1 ^biotin and 10 μg ml^-1 ^thiamine (both filter sterilized). MMP contains the same components plus 1 μM calcium pantothenate. To determine growth rates on MM or MMP, *Rhizobium *strains were grown to saturation in PY medium, the cells were harvested by centrifugation, washed twice with sterile deionized water and diluted to an initial optical density of 0.05 at 600 nm (OD_600_) when added to 30 ml of MM. These cultures were grown for 24 h in 125 ml Erlenmeyer flasks to deplete any endogenous pantothenate. Cells were then harvested and washed as described above and added to fresh MM or MMP in the same manner as for the first inoculation and cultured for 15 hours. Bacterial growth was quantified by measuring optical density at 600 nm (OD_600_) every 3 hours. Antibiotics were used at the following concentrations (in μg ml^-1^): chloramphenicol (Cm), 30; tetracycline (Tc), 10; kanamycin (Km), 30; gentamicin (Gm), 30; spectinomycin (Sp), 100; nalidixic acid (Nal), 20. *E. coli *transformants harboring recombinant plasmids (β-galactosidase-positive) were identified by growth on LB plates with 30 μg ml^-1 ^5-bromo-4-chloro-3-indolyl-β-D-galactoside (X-Gal).

### DNA manipulations

Standard techniques described by Sambrook *et al. *[28] were used for plasmid and total DNA isolation, restriction, cloning, transformations, and agarose gel electrophoresis. Plasmid mobilization from *E. coli *to *Rhizobium *was done by conjugation performed on PY plates at 30°C by using overnight cultures grown to stationary phase. Donors (*E. coli *strain S17-1) and recipients (*R. etli *CFN42 wild type and mutant strains) were mixed at a 1:2 ratio, and suitable markers were used for transconjugant selection.

### Mutagenesis of the *panC *and *panB *genes and genetic complementation of mutant strains

Mutants were generated by site-directed vector integration mutagenesis. Internal 400 bp DNA fragments of *panC *and *panB *were amplified by PCR with primers A and B; C and D, respectively (Table 3). PCR fragments of *panC *and *panB *were cloned in vector pBC as 400 bp *BamH*I-*Xba*I fragments, generating pBC1 and pBC2 respectively, and then subcloned as *Kpn*I-*Xba*I fragments into suicide vector pK18mob [29] to form plasmids pTV1 and pTV2, respectively. These plasmids were mobilized into *R. etli *CFN42 by conjugation and single crossover recombinants selected on PY plates containing Km and Nal. The disruption of the *panC *and *panB *genes was confirmed by Southern blot analysis using a 400-bp PCR internal fragment of each gene as a probe (data not shown). The resultant mutants were named ReTV1 and ReTV2 respectively. To complement the phenotype of the *panC *and *panB *mutants, plasmids pTV4, pTV5, pTV6 and pTV7 were constructed as follows: a 3.1 kb *Eco*RI fragment from cosmid vector pCos24, isolated from a genomic library of *R. etli *CFN42 [30] and containing the *panC *and *panB *genes, was subcloned in broad-host-range vector pRK7813, generating plasmid pTV4. To construct plasmid pTV5, a 1.2 kb fragment containing only *panC *(894 bp) was amplified by PCR with primers E and F and cloned in the *KpnI*-*Xba*I sites in the broad-host-range vector pBBRMSC3 so that the gene would be constitutively expressed from the vector's *lacZ *promoter. Primers G and H (Table 3) were used to amplify a 1 kb PCR fragment containing only the *panB *gene (822 bp). This DNA fragment was cloned in plasmid pBBRMSC3 in the *KpnI*-*Xba*I restriction sites, generating plasmid pTV6. Plasmid pTV7 contains the second *panB *gene (RHE_PE00443), encoded on *R. etli *plasmid p42e, this gene was amplified with primers I and J. The resultant 1 kb PCR fragment was cloned in the *KpnI*-*Xba*I sites of plasmid pBBRMSC3. To complement the growth deficiency of strain CFNX186, a derivative of *R. etli *CFN42 cured of plasmid p42f, plasmid pTV4 and cosmid vector pCos24 were introduced by conjugation. The complemented strains obtained were named CFNX186-4 and CFNX186-24 respectively. The *argE *gene was disrupted as described above. Briefly, an internal 400 bp PCR fragment of *argE *amplified with primers K and L was cloned directly in pK18mob using the *Kpn*I and *Xba*I sites to give pTV3 (Table 1). This recombinant suicide plasmid was mobilized into *R. etli *CFN42 and the resultant mutant named ReTV3 (Table 1).

**Table 3:** Primers used in this work.

Primer	Sequence (5'- 3')
A	GC**GGATCC**GAAGACCTCAGCAAATACCCGC
B	CGGA**GGATCC**GCGCCACGACGACCGACCCGCC
C	CGGG**TCTAGA**CTCGGCATGGTGCTCTATGGCA
D	GACG**TCTAGA**GCTTGAAATCGTTGAAGAGCCC
E	TGAT**GGTACC**TTGACGGATGGGGCAATAGCGG
F	GGCGC**TCTAGA**ATCCGATGGCGCTCATTTCG
G	GCGGGC**GGTACC**AGCCGGGAAAGGGAGTG
H	AAGCG**TCTAGA**GCCTTCGTCTTACGGCCG
I	CGTCAA**GGTACC**ATCCCTTCTGACCGCCTG
J	CCCCC**TCTAGA**CGCTGGGGAGAAGGGACTC
K	GCTGT**GGTACC**CGCCGTCCCGGCACTCGCG
L	ACCCT**TCTAGA**TGCCGACCTGGAGGGAGG

The restriction sites are indicated in bold.

### Filter blots hybridization and plasmid visualization

For Southern-type hybridizations, genomic DNA was digested with appropriate restriction enzymes, electrophoresed in 1% (w/v) agarose gels, blotted onto nylon membranes, and hybridized under stringent conditions, as previously reported by [31], using Rapid-hyb buffer. To use the *panC *and *panB *genes as probes, both genes were amplified by PCR, separated on a 1% agarose and purified by a PCR purification kit (QIAquick). They were labeled with [α-32P]dCTP using a Rediprime DNA labeling system. Plasmid profiles were visualized by the Eckhardt technique as modified by [21], and hybridized in a similar manner.

### Identification of orthologous proteins, multiple sequence alignments and phylogenetic analysis

All genomic sequences analyzed in this study were obtained from the Integrated Microbial Genomes System of the DOE Joint Genome Institute http://img.jgi.doe.gov/). We obtained protein and gene sequences of *panB, panC *and 10 chromosomal housekeeping genes (*fusA, guaA, ileS, infB, recA, rplB, rpoB, rpoC, secY and valS*) from 16 rhizobial species. Accession numbers for these sequences and the species list are shown in Table S1 (see Additional file 1). An orthologous data set for each gene was constructed using Blast [32] and the bidirectional best hit method applying the criteria reported by Poggio *et al *[33]. Multiple alignments of putative orthologous proteins were performed using the MUSCLE program [34] with default settings. After removing poorly conserved regions two concatenated protein alignments were obtained, one for the 10 chromosomal housekeeping genes (8469 amino acids) and the other for *panB *and *panC *(659 amino acids). Both concatenated protein multiple alignments were used to generate nucleic acids multiple alignments of their respective genes with the Tranalign program of the EMBOSS suit http://emboss.sourceforge.net/. Nucleic acids multiple alignments were used to obtain two phylogenies with the maximum likelihood method implemented in PHYML [35] with HKY as substitution model [36]. The phylogenetic reconstruction was carried out with a nonparametric bootstrap analysis of 100 replicates for each alignment. TreeDyn program [37] was used to visualize and edit both phylogenies.

## List of abbreviations

PBAL: pantoate-β-alanine ligase; MOHMT: 3-methyl-2-oxobutanoate hydroxymethyltransferase; MM: minimal medium.

## Authors' contributions

TV designed and constructed all the mutants, did all the experiments for genetic complementation of the mutants, performed growth experiments and Southern blot hybridizations and helped to draft the manuscript. SB provided intellectual guidance and contributed to writing the manuscript. AD performed Eckhardt gels and Southern blot to localize *panCB *homologues in plasmids of *R. etli *strains and assisted in DNA cloning. LL carried out the phylogenetic analysis and the discussion of results. DR participated in the experimental design and in the discussion of results. AGS conceived the study, supervised the experimental work and wrote the manuscript. All authors read and approved the final manuscript.

## Supplementary Material

Additional file 1 **Table S1**. Rhizobial species list and accession numbers of housekeeping and *panCB *genes used for phylogenetic analysis. Click here for file

## References

[B1] Jumas-Bilak E Michaux-Charachon S Bourg G Ramuz M Allardet-Servent A Unconventional genomic organization in the alpha subgroup of the Proteobacteria J Bacteriol 1998 180 2749 2755 9573163 10.1128/jb.180.10.2749-2755.1998 PMC107230

[B2] MacLean AM Finan TM Sadowsky MJ Genomes of the symbiotic nitrogen-fixing bacteria of legumes Plant Physiol 2007 144 615 622 10.1104/pp.107.101634 17556525 PMC1914180

[B3] Romero D Brom S Phillips G, Funnell BE The symbiotic plasmids of the *Rhizobiaceae* Plasmid biology 2004 Washington, D.C: American Society for Microbiology 271 290

[B4] Young JP Crossman LC Johnston AW Thomson NR Ghazoui ZF Hull KH Wexler M Curson AR Todd JD Poole PS Mauchline TH East AK Quail MA Churcher C Arrowsmith C Cherevach I Chillingworth T Clarke K Cronin A Davis P Fraser A Hance Z Hauser H Jagels K Moule S Mungall K Norbertczak H Rabbinowitsch E Sanders M Simmonds M Whitehead S Parkhill J The genome of *Rhizobium leguminosarum *has recognizable core and accessory components Genome Biol 2006 7 R34 10.1186/gb-2006-7-4-r34 16640791 PMC1557990

[B5] Crossman LC Castillo-Ramírez S McAnnula C Lozano L Vernikos GS Acosta JL Ghazoui ZF Hernández-González I Meakin G Walker AW Hynes MF Young JPW Downie JA Romero D Johnston AWB Dávila G Parkhill J González V A common genomic framework for a diverse assembly of plasmids in the symbiotic nitrogen fixing bacteria PLoS ONE 2007 3 e2567 10.1371/journal.pone.0002567 PMC2434198 18596979

[B6] González V Santamaria RI Bustos P Hernández-González I Medrano-Soto A Moreno-Hagelsieb G Janga SC Ramírez MA Jimenez-Jacinto V Collado-Vides J Dávila G The partitioned *Rhizobium etli *genome: genetic and metabolic redundancy in seven interacting replicons Proc Natl Acad Sci USA 2006 103 3834 3839 16505379 10.1073/pnas.0508502103 PMC1383491

[B7] Bittner AN Foltz A Oke V Only one of five *groEL *genes is required for viability and successful symbiosis in *Sinorhizobium meliloti* J Bacteriol 2007 189 1884 1889 10.1128/JB.01542-06 17158666 PMC1855696

[B8] Rodríguez-Quiñones F Maguire M Wallington EJ Gould PS Yerko V Downie JA Lund PA Two of the three *groEL *homologues in *Rhizobium leguminosarum *are dispensable for normal growth Arch Microbiol 2005 183 253 265 15830189 10.1007/s00203-005-0768-7

[B9] Yost CK Rath AM Noel TC Hynes MF Characterization of genes involved in erythritol catabolism in *Rhizobium leguminosarum bv. viciae* Microbiology 2006 152 2061 2074 10.1099/mic.0.28938-0 16804181

[B10] Finan TM Weidner S Wong K Buhrmester J Chain P Vorhölter FJ Hernandez-Lucas I Becker A Cowie A Gouzy J Golding B Pühler A The complete sequence of the 1,683 kb pSymB megaplasmid from the N2- fixing endosymbiont *Sinorhizobium meliloti* Proc Natl Acad Sci USA 2001 98 9889 9894 10.1073/pnas.161294698 11481431 PMC55548

[B11] Charles TC Finan TM Analysis of a 1600-Kilobase *Rhizobium meliloti *megaplasmid using defined deletions generated *in vivo* Genetics 1991 127 5 20 1849856 10.1093/genetics/127.1.5 PMC1204311

[B12] Cheng J Sibley CD Zaheer R Finan TM A *Sinorhizobium meliloti **minE *mutant has an altered morphology and exhibits defects in legume symbiosis Microbiology 2007 153 375 387 10.1099/mic.0.2006/001362-0 17259609

[B13] Harrison PW Lower RPJ Kim NKD Young JPW Introducing the bacterial "chromid": not a chromosome, not a plasmid Trends Microbiol 2010 18 141 148 10.1016/j.tim.2009.12.010 20080407

[B14] Jackowski S Neidhardt FC, Curtiss R III, Ingraham JL, Lin ECC, Low KB, Magasanik B, Reznikoff WS, Riley M, Schaechter M, Umbarger HE Biosynthesis of pantothenic acid and coenzyme A Escherichia coli and Salmonella: cellular and molecular biology 1996 Washington, DC: ASM Press 1310 1324

[B15] González V Acosta JL Santamaría RI Bustos P Fernández JL Hernández IL Díaz R Flores M Palacios R Mora J Dávila G Conserved symbiotic plasmid DNA sequences in the multireplicon pangenomic structure of *Rhizobium etli* Appl Environ Microbiol 2010 76 1604 1614 20048063 10.1128/AEM.02039-09 PMC2832352

[B16] Koonin EV Makarova KS Aravind L Horizontal gene transfer in prokaryotes: quantification and classification Annu Rev Microbiol 2003 55 709 742 10.1146/annurev.micro.55.1.709 PMC4781227 11544372

[B17] Slater SC Goldman BS Goodner B Setubal JC Farrand SK Nester EW Burr TJ Banta L Dickerman AW Paulsen I Otten L Suen G Welch R Almeida NF Arnold F Burton OT Du Z Swing A Godoy E Heisel S Houmiel KL Jhaveri J Lu J Miller NM Norton S Chen Q Phoolcharoen W Ohlin V Ondrusek D Pride N Stricklin SL Sun J Wheeler C Wilson L Zhu H Wood DW Genome sequences of three *Agrobacterium biovars *help elucidate the evolution of multichromosome genomes in bacteria J Bacteriol 2009 191 2501 2511 10.1128/JB.01779-08 19251847 PMC2668409

[B18] Brom S Garcia-de los Santos A Stepkowski T Flores M Dávila G Romero D Palacios R Different plasmids of *Rhizobium leguminosarum *bv. phaseoli are required for optimal symbiotic performance J Bacteriol 1992 174 5183 5189 1644746 10.1128/jb.174.16.5183-5189.1992 PMC206350

[B19] Vargas MC Encarnacion S Davalos A Reyes-Perez A Mora Y Garcia-de los Santos A Brom S Mora J Only one catalase, KatG, is detectable in *Rhizobium etli*, and is encoded along with the regulator OxyR on a plasmid replicon Microbiology 2003 149 1165 1176 10.1099/mic.0.25909-0 12724378

[B20] Xu Y Labedan B Glansdorff N Surprising arginine biosynthesis: a reappraisal of the enzymology and evolution of the pathway in microorganisms Microbiol Mol Biol Rev 2007 71 36 47 10.1128/MMBR.00032-06 17347518 PMC1847373

[B21] Hynes MF McGregor NF Two plasmids other than the nodulation plasmid are necessary for formation of nitrogen-fixing nodules by *Rhizobium leguminosarum* Mol Microbiol 1990 4 567 574 10.1111/j.1365-2958.1990.tb00625.x 2161988

[B22] Dehal PS Joachimiak MP Price MN Bates JT Baumohl JK Chivian D Friedland GD Huang KH Keller K Novichkov PS Dubchak IL Alm EJ Adam PA MicrobesOnline: an integrated portal for comparative and functional genomics Nucleic Acids Res 2010 38 396 400 10.1093/nar/gkp919 PMC2808868 19906701

[B23] Williams KP Sobral BW Dickerman AW A robust species tree for the *Alphaproteobacteria* J Bacteriol 2007 189 4578 4586 10.1128/JB.00269-07 17483224 PMC1913456

[B24] Guo X Flores M Mavingui P Fuentes SI Hernández G Dávila G Palacios R Natural genomic design in *Sinorhizobium meliloti*: novel genomic architectures Genome Res 2003 13 1810 1817 12902376 10.1101/gr.1260903 PMC403772

[B25] García-de los Santos A Brom S Characterization of two plasmid-borne *lpsβ *loci of *Rhizobium etli *required for lipopolysaccharide synthesis and for optimal interactions with plants Mol Plant Microbe Interact 1997 10 891 902 9304861 10.1094/MPMI.1997.10.7.891

[B26] Noel KD Sánchez A Fernández L Leemans J Cevallos MA *Rhizobium phaseoli *symbiotic mutants with transposon Tn*5 *insertions J Bacteriol 1984 158 148 155 6325385 10.1128/jb.158.1.148-155.1984 PMC215392

[B27] Encarnación S Willms K Mora J Fermentative and aerobic metabolism in *Rhizobium etli* J Bacteriol 1995 177 3058 3066 7768801 10.1128/jb.177.11.3058-3066.1995 PMC176993

[B28] Sambrook J Fitsch EF Maniatis T Molecular Cloning: A Laboratory Manual 1989 Cold Spring Harbor, Cold Spring Harbor Press

[B29] Schafer A Tauch A Jager W Kalinowski J Thierbach G Puhler A Small mobilizable multi-purpose cloning vectors derived from the *Escherichia coli *plasmids pK18 and pK19: selection of defined deletions in the chromosome of *Corynebacterium glutamicum* Gene 1994 145 69 73 10.1016/0378-1119(94)90324-7 8045426

[B30] García-de los Santos A López E Cubillas CA Noel KD Brom S Romero D Requirement of a plasmid-encoded catalase for survival of *Rhizobium etli *CFN42 in a polyphenol-rich environment Appl Environ Microbiol 2008 74 2398 2403 18310436 10.1128/AEM.02457-07 PMC2293148

[B31] Flores M González V Brom S Martinez E Piñero D Romero D Dávila G Palacios R Reiterated DNA sequences in *Rhizobium *and *Agrobacterium *spp J Bacteriol 1987 169 5782 5788 3450286 10.1128/jb.169.12.5782-5788.1987 PMC214138

[B32] Altschul S Madden T Schaffer A Zhang J Zhang Z Millar W Lipman D Gapped BLAST and PSI-BLAST: a new generation of protein database search programs Nucleic Acids Res 1997 25 3389 3402 10.1093/nar/25.17.3389 9254694 PMC146917

[B33] Poggio S Abreu-Goodger C Fabela S Osorio A Dreyfus G Vinuesa P Camarena L A complete set of flagellar genes acquired by horizontal transfer coexists with the endogenous flagellar system in *Rhodobacter sphaeroides* J Bacteriol 2007 189 3208 3216 10.1128/JB.01681-06 17293429 PMC1855832

[B34] Edgar RC MUSCLE: a multiple sequence alignment method with reduced time and space complexity BMC Bioinformatics 2004 5 113 10.1186/1471-2105-5-113 15318951 PMC517706

[B35] Guindon S Gascuel O Efficient biased estimation of evolutionary distances when substitution rates vary across sites Mol Biol Evol 2002 4 534 543 10.1093/oxfordjournals.molbev.a004109 11919295

[B36] Hasegawa M Kishino H Yano T Dating the human-ape splitting by a molecular clock of mitochondrial DNA J Mol Evol 1985 22 160 174 10.1007/BF02101694 3934395

[B37] Chevenet F Brun C Banuls AL Jacq B Chisten R TreeDyn: towards dynamic graphics and annotations for analyses of trees BMC Bioinformatics 2006 7 439 10.1186/1471-2105-7-439 17032440 PMC1615880

[B38] Piñero D Martinez E Selander RK Genetic diversity and relationships among isolates of *Rhizobium leguminosarum *biovar *phaseoli* Appl Environ Microbiol 1988 54 2825 2832 3214160 10.1128/aem.54.11.2825-2832.1988 PMC204380

[B39] Simon R High frequency mobilization of gram-negative bacterial replicons by the *in vitro *constructed Tn*5*-mob transposon Mol Gen Genet 1984 196 413 420 10.1007/BF00436188 6094969

[B40] Jones JDG Gutterson N An efficient mobilizable cosmid vector, pRK7813, and its use in a rapid method for marker exchange in *Pseudomonas fluorescens *strain HV37a Gene 1987 61 299 306 10.1016/0378-1119(87)90193-4 2833429

[B41] Kovach ME Elzer PH Hill DS Robertson GT Farris MA Roop RM II Peterson KM Four new derivatives of the broad-host-range cloning vector pBBR1MCS, carrying different antibiotic-resistance cassettes Gene 1995 166 175 176 10.1016/0378-1119(95)00584-1 8529885

