# Supplementary material for: Housekeeping genes essential for pantothenate biosynthesis are plasmid-encoded in Rhizobium etli and Rhizobium leguminosarum
Source: BMC Microbiol. 2011 Apr 5;11:66. doi: 10.1186/1471-2180-11-66 (PMC3082293; doi:10.1186/1471-2180-11-66)
Supplement: Additional file 1 — Table S1. Rhizobial species list and accession numbers of housekeeping and panCB genes used for phylogenetic analysis. [file 1471-2180-11-66-S1.DOC]

## Table S1. Rhizobial species list and accession numbers of housekeeping and panCB genes used for phylogenetic analysis.

| *Azorhizobium caulinodans* ORS571 |
| --- |
| *Agrobacterium radiobacter* K84 |
| *A.tumefaciens* C58 |
| *A. vitis* S4 |
| *Bradyrhizobium japonicum* USDA110 |
| *Bradyrhizobium sp.* BTAi1 |
| *Mesorhizobium loti* MAFF303099 |
| *Mesorhizobium sp.* BNC1 |
| *Rhizobium etli* CIAT652 |
| *R. etli* CFN42 |
| *Rhizobium sp.* NGR234 |
| *R. leguminosarum* bv. trifolii WSM2304 |
| *R. leguminosarum* bv. trifolii WSM1325 |
| *R. leguminosarum* bv. viciae 3841 |
| *Sinorhizobium medicae* WSM419 |
| *S. meliloti* 1021 |
| **Accession numbers:** |
| ***fusA*** : NP_102117 NP_772043 YP_674241 NP_354925 YP_767375 YP_469194 YP_001240980 YP_001525473 YP_001977894 YP_002280849 YP_002544203 YP_002549320 YP_002825723 YP_002975258 YP_001326671 NP_385459 |
| ***guaA*** : NP_107351 NP_770629 YP_673063 NP_353311 YP_765921 YP_467846 YP_001239609 YP_001526621 YP_001976506 YP_002283792 YP_002543108 YP_002548219 YP_002828126 YP_002978348 NP_384420 YP_001329168 |
| ***ileS*** : NP_108385 NP_774121 YP_672994 NP_353710 YP_766501 YP_468370 YP_001237831 YP_001527155 YP_001977083 YP_002279998 YP_002543541 YP_002547592 YP_002824963 YP_002974363 YP_001326104 NP_384903 |
| ***infB*** : YP_467668 YP_001976327 YP_765729 YP_002978165 YP_002283555 YP_002542855 NP_384345 YP_002828044 YP_001329094 YP_002548106 NP_353122 YP_676462 NP_106198 YP_001522936 YP_001236266 NP_767423 |
| ***recA*** : NP_101919 NP_772395 YP_673824 NP_354855 YP_768221 YP_469830 YP_001241324 YP_001525771 YP_001978551 YP_002281469 YP_002544695 YP_002549842 YP_002826165 YP_002975985 YP_001327200 NP_385905 |
| ***rplB*** : NP_102123 NP_772037 YP_674235 NP_354920 YP_767381 YP_469200 YP_001240974 YP_001525467 YP_001977900 YP_002280855 YP_002544208 YP_002549326 YP_002825729 YP_002975264 YP_001326677 NP_385465 |
| ***rpoB*** : NP_102110 NP_772050 YP_674377 NP_354931 YP_767370 YP_469189 YP_001240987 YP_001523803 YP_001977889 YP_002280844 YP_002544198 YP_002549314 YP_002825718 YP_002975253 YP_001326666 NP_385454 |
| ***rpoC***: YP_469190 YP_001977890 YP_002280845 YP_767371 YP_002975254 YP_002544199 NP_385455 YP_002825719 YP_001326667 NP_354930 YP_002549315 YP_001240986 NP_772049 YP_001523804 NP_102111 YP_674376 |
| ***secY* :** NP_102140 NP_772020 YP_674218 NP_354903 YP_767398 YP_469217 YP_001240957 YP_001525450 YP_001977917 YP_002280872 YP_002544225 YP_002549343 YP_002825745 YP_002975281 YP_001326694 NP_385482 |
| ***valS*** : NP_102766 NP_771115 YP_674132 NP_354706 YP_767633 YP_469331 YP_001239923 YP_001525731 YP_001978042 YP_002280993 YP_002544304 YP_002549938 YP_002825890 YP_002975500 YP_001326837 NP_385622  ***panB*** : NP_102046 NP_770688 YP_675108 NP_357126 YP_764870 YP_472623 YP_001239755 YP_001527247 YP_001984638 YP_002278674 YP_002544954 YP_002550059 YP_002826610 YP_002972917 YP_001327727 NP_386267 |
| ***panC*** : NP_768742 YP_675109 NP_357127 YP_764869 YP_472622 YP_001524505 YP_001984637 YP_002278675 YP_002544953 YP_002550058 YP_002826611 YP_002972916 YP_001240713 YP_001327728 NP_386268 NP_102045 |
